# Echocardiography training for cardiac surgery residents: results of a Canadian needs assessment

**DOI:** 10.1186/s13019-016-0496-x

**Published:** 2016-07-13

**Authors:** Mazin Fatani, Kevin Lachapelle, Farhan Bhanji, Peter McLeod

**Affiliations:** McGill University, Montreal, Canada; Umm Al-Qura University, Makkah, Saudi Arabia

## Abstract

**Background:**

In assessing an unstable patient post cardiac surgery, echocardiography can be an essential tool as part of this assessment. However, it may be under-utilized for several reasons. We conducted this study to  determine the perceived needs and training objectives for echocardiography training for cardiac surgery residents.

**Methods:**

This study was a cross-sectional, stratified national survey of cardiac surgery residents, cardiac surgeons, cardiac surgery program directors and cardiologists, designed to acquire opinions on what type and level of objective-based training in echocardiography is required for cardiac surgery residents. Recruitment of survey subjects was through Fluid Surveys email invitations to 201 physicians. Participants were asked to rate the importance of focused echocardiography training for cardiac surgery residents and also give a grade of importance for 18 training objectives.

**Results:**

We received 89 completed surveys. More than 80 % of responders feel that echocardiography training should be required for cardiac surgery residents. Forty seven percent of all responders felt that residents should take an echocardiography course with or without a rotation to train in echocardiography. Thirty five percent felt that current training in most programs, which entails a single rotation in echocardiography, is sufficient. Seven training objectives were identified as important by more than 80 % of participants.

**Conclusion:**

Study participants believe that cardiac surgery residents require echocardiography training. The majority agree that echocardiography training should be informed by the identified 7 training objectives.

## Background

Echocardiography is a non-invasive diagnostic tool that can provide useful information about the function and anatomy of the cardiovascular system. In unstable patients a bedside echocardiogram can guide resuscitative interventions and can be lifesaving [1–4]. Currently, many intensivists and emergentologists are adept at using focused echocardiography as an aid to dealing with challenging clinical scenarios in a timely manner. Numerous studies demonstrate high accuracy results of this diagnostic tool when done by non-cardiologists who have undergone brief training [3, 5–7]. Echocardiography can be detrimental to assessment of unstable patients on a cardiac surgery ward, especially in the hands of a trained practicing cardiac surgeons or cardiac surgery residents. Currently, the Royal College of Physicians and Surgeons of Canada specialty requirements for cardiac surgery do not mandate resident training in bedside echocardiography. In most Canadian training programs, cardiac surgery residents do a four-week rotation in an echocardiography laboratory.

Needs assessment is crucial in educational planning as education becomes linked to practice [8, 9]. Surprisingly, the learning needs for cardiac surgery residents’ training in echocardiography have never been assessed. Furthermore, we found no credible literature addressing this issue.

This pan-Canadian survey was designed to acquire a national consensus of opinions regarding the perceived echocardiography training needs of cardiac surgery residents. The target audience included cardiac surgeons, cardiac surgery program directors, cardiologists and cardiac surgery residents.

## Methods

### Study design

This study was a cross-sectional, purposive sampling, national survey of cardiac surgery residents, cardiac surgeons, cardiac surgery program directors and cardiologist educators. The survey was conducted online using FluidSurveys between March and May 2013. Study participants were recruited via email invitations. Statistical power calculations estimated that 50 subjects per group were needed in order to detect a 30 % difference in opinion with a power of 0.80 between any two groups at 0.05 significance. All 12 Canadian cardiac surgery program directors were included in the survey. The Canadian Society of Cardiac Surgeons provided the current list of 73 cardiac surgery residents who were surveyed. Since the remaining two groups are larger populations we asked each cardiac surgery program director and cardiology program director to provide a list of four surgeons and four echocardiographers respectively. Sixty five cardiac surgeons and 51 cardiologists were enrolled. The survey was anonymous and participants were sent two email reminders two weeks apart. The Institutional Review Board of the Faculty of Medicine at McGill University, Montreal, Canada approved the study.

### Measurements

In the first section of the survey, respondents were asked for demographic data including age, sex, professional role, location of practice and own perceived level of competency in echocardiography. In the second section, we asked respondents to opine whether cardiac surgery residents require training in echocardiography and if so what level of training would be required. The Canadian Society of Echocardiography identifies three levels of training based on minimum training duration and procedural volume. Level one is a minimum of four weeks of training and at least 40 echocardiography studies, level two is a minimum of six training blocks (approximately 6 months) and at least 150 complete transthoracic studies, level three is a one year fellowship [10, 11]. In the survey package participants received these definitions as guides to which level they think the residents should be trained. An echocardiography course was added to these options as another possible mode of training. In the third section of the survey, respondents were asked to rate the importance of 18 objectives by selecting one of four options for each objective (1)”must be included”, (2)“should be included”, (3) “nice to include” or (4)“should not be included”. These 18 training objectives were developed by the authors from reviewing the echocardiography training guidelines of the American and Canadian societies of echocardiography as well as objectives published in studies of focused echocardiography training for non-cardiologists. Two senior cardiologists at McGill University reviewed the list of objectives which was then pilot-tested and revised by a committee consisting of a staff cardiologist, a cardiac surgeon and three senior residents in emergency medicine and intensive care training. Participants had an opportunity to write additional objectives on the survey instrument if they wished.

## Data analysis

**Fig. 1:**
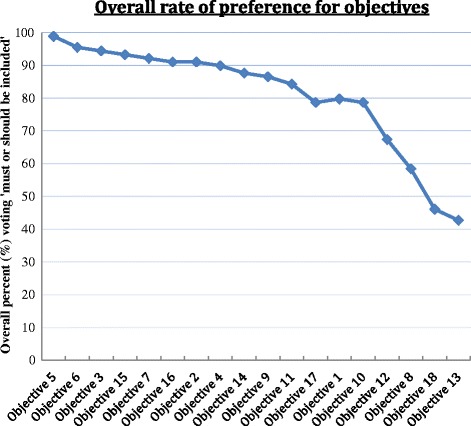
The Overall percent voting “must or should be included” for each of 18 objectives included in the survey

The study population characteristics and responses to all survey questions were characterized using descriptive statistics, including frequency and percentage. For descriptive parsimony, the five response categories for each of the 18 objectives were collapsed into three: 'must be included' or 'should be included'=’must or should be included’, 'nice to include' or 'unanswered' = neutral, 'should not be included' = should not be included. Based on a natural change in the slope of the line representing the distribution of overall votes for’must or should be included’ ordered from highest to lowest preferred objective at about 80 %, objectives that had an overall votes of 80 % or more for ‘must or should be included’ were classified as having achieved a substantial agreement (Fig. 1). We compared physician groups using chi-square and when appropriate (for smaller samples) Fisher’s exact tests for independence. Physician group comparisons included cardiac surgeons and directors combined compared to either residents or cardiologists or a specific physician group compared to everybody else for selecting a specific response such as ‘must or should be included’. All *P*-values are for two-tailed tests with statistical significance defined as *P* ≤0.05. SAS software (SAS version 9.3, SAS Institute, Inc., Cary, North Carolina) was used for all analyses.

## Results

**Fig. 2:**
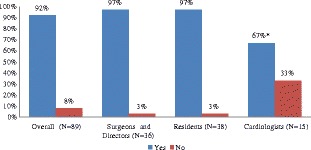
Responses of cardiac surgeons, residents and cardiologists on whether or not cardiac surgery residents require training in echocardiography

**Fig. 3:**
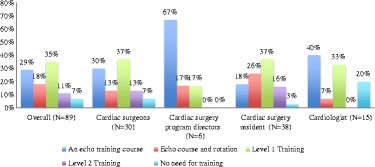
Responders’ opinion on which level of echocardiography training is required for cardiac surgery residents

**Fig. 4:**
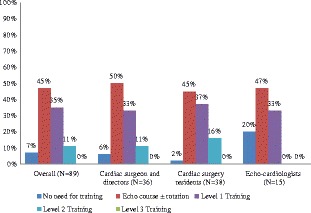
The majority in each of the 3 groups considered an echo course with or without a rotation to train echocardiography

**Fig. 5:**
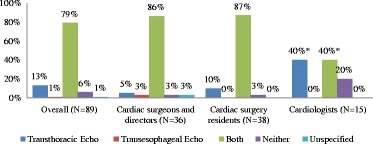
Responses on whether cardiac surgery residents should be trained in preforming transthoracic or transesophageal echo or both

Of the 201 surveys sent, 89 were completed (Table 1). Residents and program directors had a similar response rate of 50 and 52 %, respectively. Cardiac surgeons had a slightly lower response rate of 46 %. Cardiologists were the most underrepresented group with a response rate of 29 %. Nevertheless we were able to detect differences in opinion between cardiologists and other respondents. Cardiac surgery residents accounted for 42.7 % of respondents. Cardiac surgeons accounted for 33.7 %; cardiologists accounted for 16.9 % and cardiac surgery program directors represented 6.7 % of the overall study sample respondents (Table 2). Respondents from Eastern Canada accounted for 37.1 % of the study sample, while respondents from Western and Central Canada accounted for 31.5 and 29.2 %, respectively. Eighty eight percent of respondents were male. The majority were between 26 and 35 years of age and all reported a “variable” self-rated level of competency in echocardiography. Ninety two percent of responders indicated that echocardiography training should be required for cardiac surgery residents. Cardiologists, the group of specialists who were in the lowest response rate group were less enthusiastic about advocating echocardiography training for cardiac surgery residents with only 67 % of the responding cardiologists agreeing that cardiac surgery residents need training in echocardiography. In stark contrast, 97 % of residents and 97 % of surgeons and program directors advocate for echocardiographic training for cardiac surgery residents (*P* < 0.05) (Fig. 2). Overall, 29 % of all responders felt that echo course alone is sufficient while 18 % thought that an echo course along with a one-month rotation are needed (Fig. 3). Therefore 47 % of all responders felt that residents should take an echo course with or without a rotation to train in echocardiography with the majority in each of the 3 groups preferring this choice against other options (Fig. 4). However, another 35 % of all responders thought that current training in most programs, which is level one training, is sufficient. Overall, 79 % of respondents felt that the residents should be trained in both transthoracic and transesophageal echo (Fig. 5). Forty percent of cardiologists and over 80 % of participants in each of the other groups thought that training should be in both transthoracic and transesophageal echo (*P* = 0.0003). Another 40 % of cardiologists suggested that training should be in transthoracic echo only and when compared to surgeons and directors using power calculations the difference was statistically significant (*P* = 0.004). Based on the survey responses, we classified the 18 training objectives into 3 categories: (1) objectives with substantial agreement among the entire sample (≥80 % of every group agreed that the objective is important), (2) objectives with strong agreement regarding importance (≥80 %) among residents, surgeons and directors but less agreement (<80 %) among cardiologists and (3) objectives with broad response variation among the entire sample (i.e. large percentages selecting ‘must or should be included’, ‘neutral’, ‘should not be included’). Objectives rated as ‘should or must be included’ by 80 % and more of all participants are (Table 3): 1) recognition of relevant cardiac anatomy, 2) estimation of the systolic function, 3) knowledge of the indications for focused echocardiography, 4) knowledge of the limitations of focused echocardiography, 5) recognition of relevant focused findings to detect pericardial effusions, 6) recognition of relevant focused findings to detect cardiac tamponade and 7) identification of marked ventricular enlargement. Objectives with strong agreement regarding importance (≥80 %) among residents, surgeons and program directors but less agreement (<80 %) among cardiologists are (Table 4): assessment of gross wall motion abnormalities, understanding of standard ultrasound windows/planes necessary to perform focused echocardiography, recognition of presence of acute valvular regurgitation, recognition of presence of an aortic dissection, recognition of presence of mediastinal clots and knowledge of ultrasound/echo basics. Objectives with broad variation among participants regarding inclusion are listed in Table 5.

**Table 1:** Survey rate of response by group of participants

	Sent	Returned	Response rate (%)
Overall	201	89	44.3
Cardiac surgery resident	73	38	52.1
Cardiac surgeon	65	30	46.2
Cardiologist	51	15	29.4
Cardiac surgery program director	12	6	50.0

**Table 2:** Characteristics of respondents (*N* = 89)

Characteristic	N	%
Professional Role		
Cardiac surgery resident	38	42.7
Cardiac surgeon	30	33.7
Cardiologist	15	16.9
Cardiac surgery program director	6	6.7
Location		
Eastern Canada	33	37.1
Western Canada	28	31.5
Central Canada	26	29.2
Unspecified	2	2.3
Age		
< 25	2	2.3
26-35	41	46.1
36-45	17	19.1
> 45	29	32.6
Gender		
Male	78	87.6
Female	10	11.2
Unspecified	1	1.1
Self-rated level of competency with Echocardiography		
1-3 (low)	19	21.4
4-6 (intermediate)	30	33.7
7-10 (high)	37	41.6
Unspecified	3	3.4
Important aspects of respondent's echocardiography training		
N/A	28	31.5
Echo course	6	6.7
Echo course and Level 1	10	11.2
Level 1	26	29.2
Level 2	6	6.7
Level 3	13	14.6
Current residents' echocardiography training in respondent's program		
Don’t know	16	18.0
1 rotation	51	57.3
> 1 rotation	10	11.2
Other	12	13.5

**Table 3:** Objectives rated as 'must or should be included' by more than 80 % overall

Training objectives			Professional Role					
	Overall		Cardiac surgery residents (*N* = 38)		Cardiologists (*N* = 15)		Cardiac surgery directors and surgeons (*N* = 36)	
	N	%	N	%	N	%	N	%
Recognition of relevant cardiac anatomy including cardiac chambers, valves, pericardium, and aorta	88	98.9	38	100.0	14	93.3	36	100.0
Estimation of the systolic function	85	95.5	37	97.4	12	80.0	36	100.0
Knowledge of the limitations of focused echocardiography	84	94.4	37	97.4	12	80.0	35	97.2
Recognition of relevant focused findings to detect pericardial effusions	83	93.3	37	97.4	13	86.7	33	91.7
Knowledge of the indications for focused echocardiography	81	91.0	35	92.1	12	80.0	34	94.4
Recognition of relevant focused findings to detect cardiac tamponade	81	91.0	36	94.7	12	80.0	33	91.7
Identification of marked ventricular enlargement	77	86.5	33	86.8	12	80.0	32	88.9

**Table 4:** Objectives rated as 'must or should be included' by more than 80 % of all residents, surgeons and directors but not cardiologists

Training objectives			Professional Role					
	Overall		Cardiac surgery residents (*N* = 38)		Cardiologists (*N* = 15)		Cardiac surgery directors and surgeons (*N* = 36)	
	N	%	N	%	N	%	N	%
Assessment of gross wall motion abnormalities	82	92.1	35	92.1	11	73.3	36	100.0
Understanding of standard ultrasound windows/planes necessary to perform focused echocardiography	80	89.9	37	97.4	11	73.3	32	88.9
Recognition of presence of acute valvular regurgitation	78	87.6	36	94.7	11	73.3	31	86.1
Recognition of presence of an aortic dissection	75	84.3	32	84.2	11	73.3	32	88.9
Recognition of presence of mediastinal clots	71	79.8	31	81.6	9	60*	31	86.1
Knowledge of ultrasound/echo basics	70	78.7	32	84.2	7	46.67*	31	86.1

*P < 0.05 for comparisons between cardiac surgeons and directors vs. cardiologists. All *p*-values were from two-tailed Fisher's Exact Test

**Table 5:** Objectives with broad variation among participants regarding inclusion

Training objectives			Professional Role					
	Overall		Cardiac surgery residents (*N* = 38)		Cardiologists (*N* = 15)		Cardiac surgery directors and surgeons (*N* = 36)	
	N	%	N	%	N	%	N	%
Recognition of dilated aortic root and/or thoracic aorta	70	78.7	29	76.3	11	73.3	30	83.3
Use of echo guidance for pericardiocentesis	60	67.4	26	68.4	9	60.0	25	69.4
Estimation of right atrial pressure through examination of inferior vena caval compliance	52	58.4	26	68.42*	8	53.3	18	50.0
Techniques to estimate pulmonary artery pressure	41	46.1	20	52.6	7	46.7	14	38.9
Confirmation of transvenous pacing wire placement	38	42.7	19	50.0	6	40.0	13	36.1

*P < 0.05 for all comparisons (cardiac surgeons and directors vs. either residents or cardiologists). All *p*-values were from two-tailed Fisher's Exact Test

## Discussion

The results of this survey indicate that there is a substantial agreement amongst cardiac surgeons, program directors and residents that cardiac surgery residents require echocardiography training. On the other hand cardiologists appear to be significantly less enthusiastic with respect to the desirability of echocardiography training for cardiac surgery residents. One third of the responding cardiologists are of the opinion that cardiac surgery residents do not require training in echocardiography. Despite the fact that cardiologists were underrepresented in the survey, power calculations indicate that this difference in opinions was statistically significant.

Survey participants were asked their opinions on the level of echocardiography training required for cardiac surgery residents. The majority of responders in each group thought that an echo course with or without a rotation in echocardiography is desirable. Others thought that level one training alone is sufficient and none of the participants thought level three is required. Very few considered a level two training as necessary.

From the selection of 18 objectives 7 were identified as ‘must or should be included' by the majority of participants. The authors feel that any echocardiography-training curriculum for cardiac surgery residents should include these seven objectives. Six other objectives were rated as important by the surgeons and the residents but were not rated as important by the cardiologists. Surgeons may have considered those objectives as mostly related to their practical needs where cardiologists may think that some of these objectives are technically more difficult to acquire during a brief period of training.

## Conclusion

We conclude that there is a need for echocardiography training for cardiac surgery residents. An echocardiography course directed to the learning needs of the residents in performing a focused echocardiography exam should be considered. We anticipate that all residents in cardiac surgery will soon be competent to perform focused echocardiography exams on unstable patients. Focused echocardiography exams should not replace clinical judgment, the physical exam and expert echocardiography. In emergency situations residents and cardiac surgeons should be capable of performing and interpreting satisfactory echocardiogram on their patients. This study can serve as a starting point to develop an echocardiography-training curriculum for cardiac surgery residents.
